# Temporal Variations in Metabolic and Autotrophic Indices for *Acropora digitifera* and *Acropora spicifera* – Implications for Monitoring Projects

**DOI:** 10.1371/journal.pone.0063693

**Published:** 2013-05-16

**Authors:** Saskia Hinrichs, Nicole L. Patten, Anya M. Waite

**Affiliations:** School of Environmental Systems Engineering and The Oceans Institute M470, University of Western Australia, Perth, Western Australia, Australia; Universidade Federal do Rio de Janeiro, Brazil

## Abstract

Coral health indices are important components of the management assessments of coral reefs, providing insight into local variation in reef condition, as well as tools for comparisons between reefs and across various time scales. Understanding how such health indices vary in space and time is critical to their successful implementation as management tools. Here we compare autotrophic and heterotrophic coral health indices, examining specifically the temporal variation driven by the local environmental variation, at three scales (diel, daily and seasonal). We compared metabolic indices (RNA/DNA ratio, protein concentration) and autotrophic indices (Chlorophyll *a* (Chl *a*), zooxanthellae density, effective quantum yield (yield) and relative electron transport rate (rETR)) for two dominant *Acropora* species, *A. digitifera* and *A. spicifera* at Ningaloo Reef (north-western Australia) in August 2010 (austral winter) and February 2011 (austral summer). Clear seasonal patterns were documented for metabolic indices, zooxanthellae density and rETR, while cyclic diel patterns only occurred for yield and rETR, and RNA/DNA ratio. Significant daily variation was observed for RNA/DNA ratio, Chl *a* concentration, yield and rETR. Results suggest that zooxanthellae density and protein concentrations are good long-term indicators of coral health whose variance is largely seasonal, while RNA/DNA ratio and rETR can be used for both long-term (seasonal) and short-term (diel) coral monitoring. Chl *a* can be used to describe changes between days and yield for both diel and daily variations. Correlations between health indices and light history showed that short-term changes in irradiance had the strongest impact on all health indices except zooxanthellae density for *A. digitifera*; for *A. spicifera* no correlation was observed at all. However, cumulative irradiance over the several days before sampling showed significant correlations with most health indices suggesting that a time-lag effect has to be taken into account when interpreting diel variations in coral condition.

## Introduction

Reef-building corals live in symbiosis with zooxanthellae (endosymbiotic algae), which enables the coral to obtain energy through autotrophy (light-derived) as well as through heterotrophy (active uptake of particles). Photosynthesis in the symbionts of corals provide a carbon source for the coral host that is energy-rich but nitrogen-poor, while the zooxanthellae benefit from high nitrogen and phosphorus metabolic waste products of the corals [1]–[3]. The presence of these photoautotrophic symbionts within the coral tissue suggests that corals should experience large daily fluctuations in O_2_, CO_2_ and NH_4_ tension and pH driven by algal photosynthesis and coral metabolism (respiration rates) over a normal light/dark cycle [4], [5]. Indeed, oxygen concentrations in the boundary layer of corals vary over diel cycles, with an anoxic state occurring at night and supersaturation occurring in day light [6]. Lipid body formation, which is dependent upon the symbiotic status between the coral host and its symbionts also exhibits diel rhythmicity with increased lipid density and size occurring during high light periods [7]. Previous studies showed that the light/dark cycle [5] also imposed a diel cycle on algal cell division [8], [9]. Diel variations in the effective quantum yield of photosystem II (PSII) have therefore been used to assess the photosynthetic performance of PSII, which varies on a diel basis for some coral species [10], [11]. There are also diel fluctuations in relative electron transport rate, an indicator for photosynthetic activity [12].

Since metabolic rates in the coral host depend on photosynthetically derived and translocated products [1], [13], the RNA/DNA ratio and other indices of metabolic activity are correlated with light [14] and can be expected to show diel changes. The RNA/DNA ratio is related to protein synthesis [14] thus protein concentration might also express diel variations. Plankton and nitrogen concentrations in the water have also been shown to drive changes in the RNA/DNA ratio and protein concentration [15], [16] and might result in diel variations. Previous work suggests that diel cycles are set by the availability of demersal planktonic food [17], [18] and alternate sources of nitrogen [19] with high concentration of zooplankton and possible highest feeding rates during night [20], [21]. Diel variations in RNA/DNA ratios have been observed for fish and molluscs [22], [23] due to diel fluctuations in metabolic rates, food requirements and digestion times [24]. However, to our knowledge no study so far has investigated diel patterns in RNA/DNA ratio and protein concentration, or investigated which physico-chemical factors might be responsible for these changes. Mechanisms driving links between diel changes in photosynthetic activity and diel changes in metabolic indices remain largely unknown. This knowledge is important for a better understanding for how autotrophic and metabolic processes are linked with each other and the environment.

Seasonal cycles have been observed for a variety of indices describing coral metabolism such as protein concentration [16], [25] and RNA/DNA ratio [14] (as well as autotrophic indices such as effective quantum yield (yield), relative electron transport rate (rETR) [26], [27] zooxanthellae density and pigments [28], [29]. Here we investigate the importance of diel or daily variations, specifically whether these variations occur and if so, whether they occur at the scale of those observed seasonally. Previous studies investigated temporal scales over which metabolic and autotrophic indices change due to changes in water quality (minutes to weeks) [30] and thus the suitability for these indices as monitors of coral health. However, no study so far has investigated simultaneously a variety of metabolic (protein concentration, RNA/DNA ratio) and autotrophic indices (zooxanthellae density and pigment concentration, effective quantum yield and relative electron transport rate) to determine the relative importance of diel, daily and seasonal changes. This understanding is essential for future interpretation of changes in health indices. In addition, the response time of health indices to light has to be taken into account when interpreting diel variation since previous studies showed that translocation of photosynthetically derived carbon can take up to days until it is integrated in the coral tissue [31].

Our earlier work at Ningaloo Reef determined seasonal changes in health indices (Chl *a* concentration, zooxanthellae density, RNA/DNA ratio and protein concentration) for two dominant *Acropora* species as well as driving physico-chemical factors for those seasonal changes [32] and showed unexpected values for autotrophic and metabolic indices during the La Niña event. Here we determine diel and daily changes for these same two *Acropora* species for a better understanding for how useful metabolic and autotrophic indicators are for short-term as well as long-term monitoring projects. In this study we define coral health as relative physiological rates – thus our autotrophic and metabolic measurements are used as indicator for physiological changes inside the coral occurring under a range of both normal and extreme (La Niña) conditions. Measurements were taken during a normal winter season and during an extreme summer season (La Niña) and so include a wide range of normal as well as abnormal variations of physiological indices. Thus the aim of this study is to determine 1) how changes in metabolic indices and autotrophic indices occur on a diel, daily and seasonal basis for *A. spicifera* and *A. digitifera*, 2) how indices which express diel changes are correlated and 3) which physico-chemical factors are most likely to predict metabolic indices (RNA/DNA ratio and protein concentration) on a diel basis and 4) the time-lag between changes in light and the response of health indices.

## Materials and Methods

### Study Site

**Figure 1:**
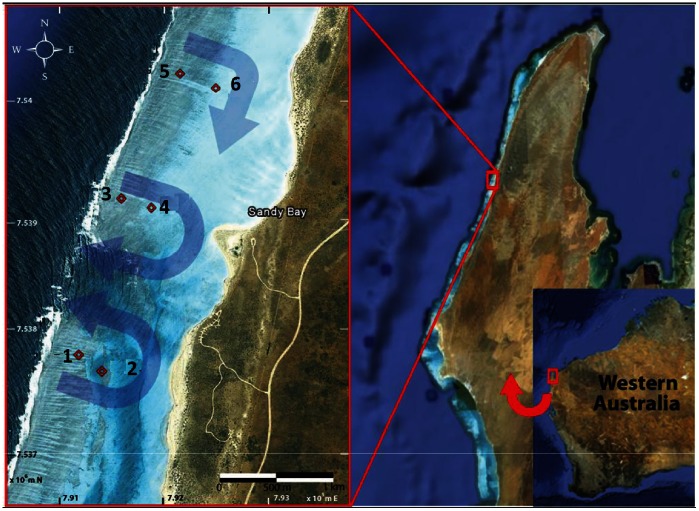
Map of the study region and sampling stations (depicted by red diamonds and numbers). Superimposed blue arrows show the characteristic flow pattern across the reef flat and around the lagoon [85]. Satellite imagery: Google Earth, 2010.

The study site, Sandy Bay Lagoon (Sandy Bay), is located at Ningaloo Reef, along the North-West Cape of Western Australia (22.23°S, 113.84°E). Sandy Bay lagoon was chosen as hydrodynamic patterns have been described previously [33], [34] and the fringing reef is typical of the ∼290 m stretch of Ningaloo Reef, with shore-parallel reef sections periodically interrupted by channels [33]. The steep reef front (∼1∶50) rises to a shallow reef crest (∼1.5 m), where waves break transporting water into the lagoon across the crest and returning back to the ocean through the channels. At Sandy Bay, the reef crest starts ∼50 m from the surf zone, spreads over ∼500 m and reaches 1000 m shoreward, giving way to a sandy lagoon habitat (depth ∼2–3 m) (Fig. 1). The dominant coral genus on the reef crest at Sandy Bay is *Acropora* [35]. Day and night sampling was conducted at one station, Station 4, which was easy accessible during most tidal levels (Fig. 1). Additionally, seasonal sampling was done at six stations (Fig. 1) on a diurnal basis to determine time-lag effects with light. Permits to conduct the field work were given by the Department of Environment and Conservation, Government of Western Australia.

### Sampling

Two different coral species, *Acoropra digitifera* and *Acropora spicifera* [36] (n = 3) were tagged at Station 4. These two corals are distinct in growth form, with *A.digitifera* a caespito-corymbose coral, and *A. spicifera*, a plate coral. Coral samples were collected four times daily (morning, noon, evening, midnight) over four days (when weather conditions were convenient) during winter (August 2010) and summer (February 2011) at Station 4 (Fig. 1). February 2011 was the peak of a strong La Niña event and therefore physico-chemical conditions were different to other summers in this region [37] with higher sea surface temperatures, comparably low light levels and high plankton and nutrient concentrations [38]. To determine time-lag effects, six coral colonies of each species (*A. digitifera* and *A. spicifera*) were tagged at six stations in the reef lagoon (Station 1–6, Fig. 1) and coral samples taken for symbiont density and Chlorophyll *a* (Chl *a*) concentration, RNA/DNA ratio, protein concentration during winter 2010, summer 2011 and additionally in autumn 2010 (March and April 2010 - before and after coral spawning). Corals were sampled while snorkelling; with *in situ* measurement on the colony for yield and rETR and removal of 1–5 cm length coral pieces from the middle of the coral colony (tip of the branch) for each of the other health indices (see below).

### Analysis of Health Indices

#### Zooxanthellae density and Chlorophyll a

Coral samples (∼3–5 cm long) for the analysis of zooxanthellae density (per cm^2^) and Chl a concentration in the tissue (µg cm^−2^) were stored at −20°C prior to analysis. Methods for zooxanthellae density and Chl a in coral tissue were adapted from Siebeck et al. [39]. Briefly, a jet of pressurized air in filtered seawater was used to remove coral tissue from the skeleton and the slurry homogenised (45 s) after measuring exact volume of the homogenate. Homogenate samples (10 ml) were taken, fixed with formaldehyde (2 ml formaldehyde per 10 ml solution) and a Neubauer haemocytometer used to count total number of zooxanthellae under a microscope (four replicate counts on four plates). Calculations of the total number of symbiotic zooxanthellae per area were based on the volume of the homogenate and the coral surface area. Coral surface area was measured using paraffin wax method [40].

Chl *a* concentration was determined following the method**s** of Jeffrey and Humphrey [41]. Briefly, three aliquots of the homogenate sample (10 ml) (as above) were centrifuged (3500×*g*) for 15 min. The supernatant was discarded and the pellet re-suspended in 10 ml of acetone (100%) and Chl *a* extracted for 24 h in the dark (−20°C). After centrifugation at 3500×*g* for 10 min fluorescence was measured in a T700 fluorometer (Turner Designs) and standardized to coral surface area.

#### RNA/DNA and protein analysis

Samples for RNA/DNA and protein analysis were stored at −80°C prior to analysis. RNA content varies with metabolic demand and is correlated with new protein synthesis, while DNA content is largely stable [14], [15]. Methods for analysis of RNA/DNA followed Humphrey [42]. Briefly, coral samples (∼1 cm long) were crushed in liquid nitrogen and TE extraction buffer (Tris-EDTA with 1% sarcosyl added (10 ml)). Samples were sonicated in an ice bath, centrifuged (1200×g for 3 min) and the supernatant (100 µl) as well as TE buffer (900 µl) transferred into deep well plates. The same solution was used for protein analysis. Samples (75 µl, triplicates), nucleic acid standards (0–2.5 µg ml^−1^ for DNA and RNA, duplicates) and control homogenates were added to three 96 deep-well microplates. Plate one was treated with TE buffer (15 µl) while plate two with TE buffer (7.5 µl) and RNAse (7.5 µl) and plate three with RNAse (7.5 µl) and DNAse (7.5 µl). Plates were incubated (35 min for plate one and two and 60 min for plate three) and Ribogreen added to each well. Plates were read in a microplate reader (485 nm excitation and 528 nm emission). RNA was calculated by subtracting the fluorescence reading of plate two from plate one while DNA was calculated by subtracting the fluorescence reading of plate three from plate two. Calculating of RNA and DNA concentrations was based on the standard curves of each plate. Finally the ratio between DNA and RNA concentration was determined.

For protein analysis, a standard DC Protein Kit was used [43]. Sample solution (5 µl, as above (triplicates)) was added together with protein standards (0.2 mg ml^−1^ to 1.5 mg ml^−1^ protein duplicates) in a 96 deep-well microplate. After adding 25 µl of reagent A and 200 µl of reagent B to each well, absorbance was read after 15 min at 750 nm. Protein content was calculated based on standard curve of each plate and all protein concentrations standardized afterwards to DNA values.

#### Effective quantum yield and relative electron transport rate

Variations in the effective quantum yield (yield) of photosystem II (PSII), a way to assess the photosynthetic performance of PSII, were measured using an underwater pulse-amplitude modulated (PAM) fluorometer (Diving-PAM, Walz) on 10 sections of the tagged coral colony. The fiber was placed at a fixed distance (1 cm) in front of the coral tissue. The yield (ΔF/Fm′) was measured by exposing 10 sections of the colony separately to a 0.8 s period of saturating light (ca. 8000 µmol m^−2^ s^−1^) [11]. Yield measurements were converted to relative eletron transport rate (rETR) with the formula rETR = ΔF/Fm′×PAR×0.5 with PAR as the immediate radiance and 0.5 as the factor that accounts for the distribution of electrons between photosystem I and photosystem II [44]. Relative measure was used for the rate of electron transport since light absorption characteristics of tissue are unknown for these species.

### Analysis of Physical, Chemical and Biological Parameters of the Water

Physico-chemical factors known to be essential for coral health were sampled at each station during the sampling period: light, temperature, current speed, nutrients (dissolved inorganic nitrogen), phytoplankton and zooplankton.

#### Abiotic factors

Light and temperature were measured using temperature loggers (Hobo Pendant Data Logger) that were deployed between 3 to 7 days before the sampling.

Short-term current speed was estimated using drifters. Two crucifix design drifters [34] containing a GPS were simultaneously deployed for approximately 10 minutes and the GPS location and time of their deployment and collection recorded. The distance and time of each deployment allowed a simple estimate of surface flow speed.

#### Biotic factors

Water quality samples were taken for the analysis of dissolved nutrients, total nitrogen (TN), Chl *a*, and picoplankton (*Synechoccocus*, *Prochloroccocus* and picoeukaryotes).

For the analysis of dissolved inorganic nitrogen (DIN) (NO_x_ (NO_3_+ NO_2_) and NH_4_), water samples (40 ml) were filtered through 0.45 µm filters and stored at −20°C, before flow injection analysis (FIA) with detection by absorbance at specific wavelengths for nitrate (NO_3_)/nitrite (NO_2_) (QuikChem FIA+Lachat 8000 series). Briefly, Nitrate was reduced to nitrite through a copperized cadmium column that than reacted with sulphanilamide under acid conditions to form a diazonium ion. The diazonium ion is coupled with N- (1-naphthyl) ehthylenediamine dihydrogenchloride that has an absorbance maximum at 520 nm (Quikchem Method 31-107-04-1-A) [45]. Ammonium was measured by fluorescence (Global FIA high sensitivity gas diffusion unit- HPMSD, Shimadzu RF-10Axl Fluorescence detector) [46]. Briefly, Ammonium was liberated as ammonia by sodium hydroxide and passed subsequently through a porous PTFE membrane (HPMSD), reacted with ortho-phthaldiadehyde and sulphite and formed a fluorescent derivative. The derivative was measured with an excitation wavelength of 310 nm and an emission wavelength of 390 nm. Dissolved inorganic nitrogen (DIN) is the sum of ammonium and nitrate and nitrite.

For total nitrogen (TN), unfiltered water samples (50 ml) were stored at −20°C until analysis. TN was determined from autoclave digests with potassium persulphate (Lachat Quick-Chem 8500 Automated Flow Injection Analyser) [47].

Organic nitrogen (ON) was calculated by subtracting DIN from TN.

Seawater samples (1 L) for Chl *a* was filtered onto 0.7 µm filters (Whatman GF/F) and the filters stored at −20°C in the dark until fluorometric analysis of duplicated 90% acetone extracts was carried out [48].

To determine concentrations of autotrophic picoplankton groups (*Synechoccocus*, *Prochlorococcus* and picoeukaryotes), 1.5 ml seawater was fixed with gluteraldehyde (0.5% final concentration) for 10 minutes and then snap frozen in liquid nitrogen. Samples were analysed using flow cytometry following the technique of Patten et al. [49]; samples were thawed at 37°C, 1 µm fluorescent beads (Molecular Probes) added as an internal standard and samples were analysed using a FACSCANTO II (Becton-Dickinson) flow cytometer fitted with a 488 nm laser on high throughput mode at a flow rate of 60 µl min^−1^ for 100 s. Nitrogen (N) and carbon (C) content of picoplankton was calculated based on fixed factors for picoeukaryotes (39.2 fg N and 836 fg C ) [50], [51], *Prochloroccocus* (4 fg N and 46 fg C) and *Synecoccocus* (30 fg N and 213 fg C) [52], [53]. Total nitrogen and carbon content for picoplankton was then calculated by the sum of nitrogen and carbon concentrations for *Prochloroccocus*, *Synechoccocus* and picoeukaryotes.

Zooplankton was sampled with a plankton net (90 cm diameter, 10 min in the water with a speed of 2.5 km h^−1^ for 50 m around the station) and preserved with formaldehyde (ca. 5%). Dry weight analyses were carried out for zooplankton between 100 and 1000 µm. Wet zooplankton samples were added to a pre-weighed container after cleaning samples of salt with DI water (30 s) and dried in an oven (60°C for 24 hours). Samples were then cooled down in a desiccator and weighted for subsequent calculation of dry weight (DW) [54].

### Statistical Analysis

A four-factor PERMANOVA was performed in PRIMER Version 6 (PRIMER-E, Plymouth, UK) with species, seasons and times per day as fixed terms and days nested in season (crossed factors) [55], [56]. PERMANOVA was based on Euclidean distances of protein concentration, RNA/DNA ratio, Chl *a* per cm^2^ and per cell, zooxanthellae density, yield and relative ETR (all log-transformed) and analysed with Type I (sequential) sum of squares with permutation of residuals under a reduced model (9999 permutations). Health indices, which showed diel patterns were in addition tested for day/night differences (morning and noon combined and evening and midnight combined) with the same design as described above but night/day instead of times per day. Log-transformation was done based on results of a draftman plot as well as Grubbs’ test to reduce outliers and make data more continuous. Pair-wise tests were performed when significant differences occurred. To determine correlations between health indices, that displayed diel patterns the coefficient of determination R^2^ was tested with DistLM (Primer 6) based also on Euclidean dissimilarity matrix after log-transformation (with selection criteria all specified).

To determine which physico-chemical factors best explain changes in protein concentration and RNA/DNA ratio on a diel basis, health indices and physico-chemical factors (light, temperature, current speed, Chl *a* concentration in the water, nitrogen and carbon content of picoplankton, zooplankton (dry weight) and DIN and ON were tested with DistLM (Primer 6) based on Bayesian information criterion (BIC) (forward procedure, 9999 permutations) [55]. Protein concentration and RNA/DNA ratio were log-transformed while physico-chemical factors were square root transformed. Correlations were done for species and seasons separately.

Correlation coefficients (R) between health indices and light history, were determined using DistLM (Primer 6) based on Euclidean dissimilarity matrix after log-transformation of health indices for integrated light during 0, 1, 3, 6, 12, 24, 36 and 48 hours prior to sampling (with selection criteria all specified). Diel as well as seasonal data was included to get a broader light spectrum.

## Results

### Temporal Variation of Health Indices

**Figure 2:**
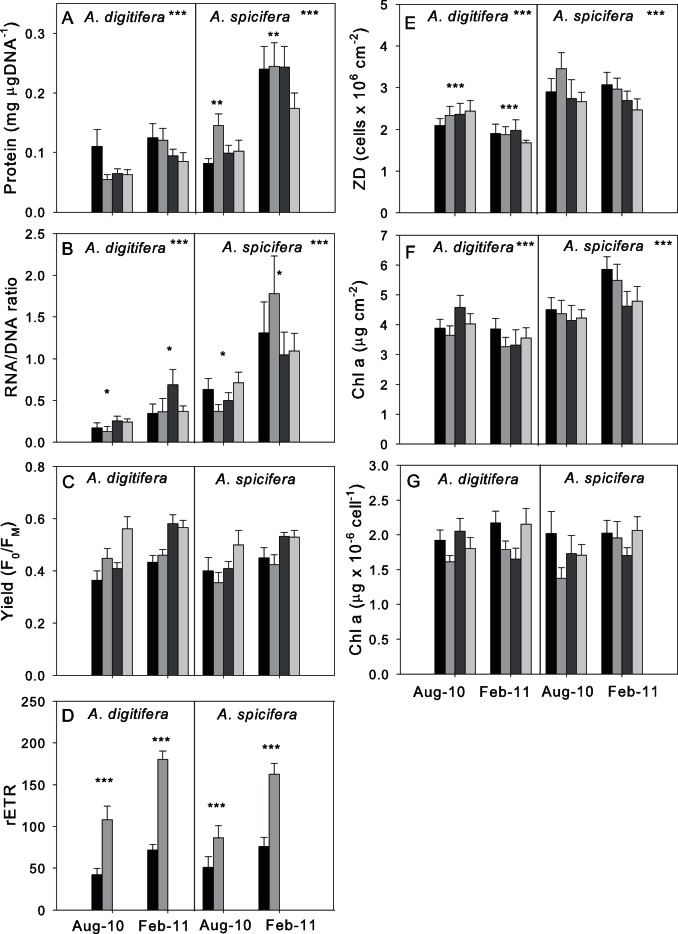
Diel pattern for health indices for *Acropora digifera* and *Acoropra spicifera* at Ningaloo Reef. Values represent Means ± Standard Error (SE), n = 9–12 for metabolic indices (protein concentration, RNA/DNA ratio) and autotrophic indices (zooxanthellae density, Chlorophyll *a* concentration (per surface area and per cell), yield and rETR)) at four different times per day in August 2010 and February 2011. Black bar = morning (6–7 am), mid grey bar = noon (12–1 pm), dark grey bar = evening (6–7 pm), light grey bar = midnight (12–1 am). Stars symbolize significant differences between species (next to coral names) and seasons (above bars) (*P<0.05; **P<0.01; ***P<0.001).

Differences between species, seasons, days and diel patterns (morning, noon, evening, midnight) were tested for *A. digitifera* and *A. spicifera* between summer 2011 (February) and winter 2010 (August) (Table S1). Diel patterns are displayed in Fig. 2.

Significant differences between species (sp) and seasons (se) were found for protein concentration (sp: F_1, 179_ = 58.22, p<0.001; se: F_1, 179_ = 27.44, p<0.01; sp×se: F_1, 179_ = 2.41, p<0.01), RNA/DNA ratio (sp: F_1, 178_ = 81.83, p<0.001, se: F_1, 178_ = 4.87, p<0.001) and zooxanthellae density (sp: F_1, 179_ = 58.22, p<0.001; se: F_1, 183_ = 4.94, p<0.05). However, pair-wise tests for protein concentration revealed that only *A. spicifera* showed seasonal differences (p<0.01) with higher protein concentrations in summer than in winter, while for zooxanthellae density significant differences were only found for *A. digitifera* (p<0.001) based on higher values during winter (Fig. 1) compared with summer. RNA/DNA ratios showed seasonal differences for both *A. digitifera* and *A. spicifera* (sp×se: F_1, 178_ = 6.34, p<0.05; pair-wise comparison p<0.05 for both) with highest values occurring during summer compared to winter for both species (Fig. 2).

Chl *a* concentration per surface area also varied significantly between species (sp: F_1, 183_ = 91.98, p<0.001) due to higher concentrations in summer for *A. spicifera* than for *A. digitifera* (sp×se: F _7, 183_ = 51.33, p<0.001, pair-wise comparison p<0.01) while no differences occurred in winter between species for Chl *a* concentration per surface area (p>0.05) (Fig. 2). No seasonal patterns were observed when analysing species individually. No significant differences between the two *Acropora* species were found for Chl *a* concentration per cell, yield and rETR (p>0.05), with only rETR expressing seasonal differences with higher rates during summer (F_1, 164_ = 28.83, p<0.001) (Fig. 2).

Protein concentration and zooxanthellae density did not significantly vary over diel or daily time scales for any of the two species. In contrast, RNA/DNA ratio (F _7, 178_ = 10.53, p<0.001), Chl *a* (Chl *a* per cm^2^: F _7, 183_ = 6.89, p<0.001; Chl *a* per cell: F _7, 183_ = 3.21, p<0.01), yield (F _7,167_ = 11.40, p<0.001) and rETR (F _7, 164_ = 17.23, p<0.001) exhibited significant differences between days within each season. Significant changes throughout the day were observed for yield as well as rETR (F _3, 167_ = 3.10, p<0.05; F _3, 167_ = 413.57, p<0.001) even though this pattern was not consistent throughout all days (da (se) ×di: F_13, 167_ = 7.55, p<0.001; F_13, 164_ = 34.37, p<0.001) (Table S2). In general for yield, evening and midnight values were higher than morning and midday values (Fig. 2) and pair-wise tests revealed that morning yield values differed significantly from those when species were pooled (p<0.05). rETR displayed the opposite pattern with highest rates occurring during midday and zero values occurring during evening and night due to a lack of light (PAR = 0) (Fig. 2). Significant differences in rETR occurred when data was pooled between all times with the exception of midnight and evening (since both zero values)) (p<0.05). When morning and noon values were combined as well as evening and midnight values day/night patterns still occurred for yield values (F _4, 167_ = 5.04, p<0.001) even though day/light variations were only apparent for some and not all days. For rETR, daily variation lost importance when testing day/night differences with day/night differences occurring for summer and winter (F_1, 167_ = 13.19, p<0.05; pair-wise comparison p<0.001). For RNA/DNA, diel patterns were observed within a day but without a consistent pattern (i.e. changing between days) (Table S3). However, when species were investigated separately, no diel difference could be resolved (sp×da(se) ×di p>0.05). In addition, when diurnal and nocturnal values were pooled, there was no significant day/night variation (p>0.05).

Correlations between health indices which changed on short-term basis showed that Chl *a* per cell was positively correlated with yield values (r = 0.17, p<0.05) and yield was also positively correlated with rETR (r = 0.27, p<0.001). R-values increased when only those values measured during day were taken into account and night values excluded (r = 0.34, p<0.01; r = 0.54, p<0.001). No correlation was found between RNA/DNA ratio with Chl *a* per cell, yield or rETR for either *A. digitifera* or *A. spicifera* within the whole day or only during daytime.

### Environmental Predictors for Metabolic Indices on a Diel Basis

Physico-chemical factors (light, temperature, current speed, DIN, ON, Chl *a* concentration, zooplankton, N-content of picoplankton and C-content of picoplankton) were tested for best prediction of coral metabolic indices (protein concentration, RNA/DNA ratio), separately for *A. digitifera* and *A. spicifera* during winter and summer. During winter changes in protein concentration during the day for *A. digitifera* were correlated with ON in the water, while RNA/DNA ratio showed strongest yet non-significant relationship with light. In summer, for *A. digitifera*, protein concentrations were not correlated with temperature or light, while RNA/DNA ratio were best explained by nitrogen provided by picoplankton and organic nitrogen (Table 1).

**Table 1:** Physico-chemical predictors for variation in protein concentration and RNA/DNA ratio in *A. digitifera* and *A. spicifera* during winter (August 2010) and summer (February 2011).

Season	Health indices	Predictor	BIC		Pseudo-F	% variability	% total
	***A. digitifera***						
Winter	Protein (mg µgDNA^−1^)	**ON (µmol l** ^−**1**^ **)** *	−271.73	6.59		13.6	13.6
	RNA/DNA ratio	*PAR (µE m* ^−*2*^ * s* ^−*1*^ *)*	−163.92	3.85		8.4	8.4
Summer	Protein (mg µgDNA^−1^)	*Temp*	−250.74	3.43		7.7	
		*DIN (µmol l* ^−*1*^ *)*	−250.86	3.78		8.0	15.7
	RNA/DNA ratio	**Pico N (fg µl** ^−**1**^ **)** *	−108.42	7.38		15.3	
		**ON (µmol l** ^−**1**^ **)** *	−108.93	4.18		8.0	23.3
	***A. spicifera***						
Winter	Protein (mg µgDNA^−1^)	Zooplankton (mg l^−1^) *	−278.08		3.99	8.1	
		PAR (µE m^−2^ s^−1^) *	−278.67		4.36	8.3	16.4
	RNA/DNA ratio	**Temp** **	−139.01		7.89	14.9	
		Pico C (fg µl^−1^) *	−139.7		4.46	7.8	22.8
Summer	Protein (mg µgDNA^−1^)	*PAR (µE m* ^−*2*^ * s* ^−*1*^ *)*	−200.76		1.23	2.9	2.9
	RNA/DNA ratio	**Chl ** ***a*** ** (µg l** ^−**1**^ **)** *	−79.198		10.66	20.6	20.6

Results are based on BIC tests (Primer). Bold letters symbolize negative correlations while other correlations were positive.

* p<0.05,

** p<0.01,

*** p<0.001.

Non-significant correlations are in italic.

For *A. spicifera*, zooplankton and light were strongly (positively) correlated with protein concentrations, while temperature was negatively, and carbon supplied by picoplankton positively correlated with variability in RNA/DNA ratio. In summer light showed strongest but non-significant correlation with protein concentration while Chl *a* concentration in the water column showed a negative correlation with RNA/DNA ratio of *A. spicifera* (Table 1).

### Time-lag Effect of Light on Health Indices

**Figure 3:**
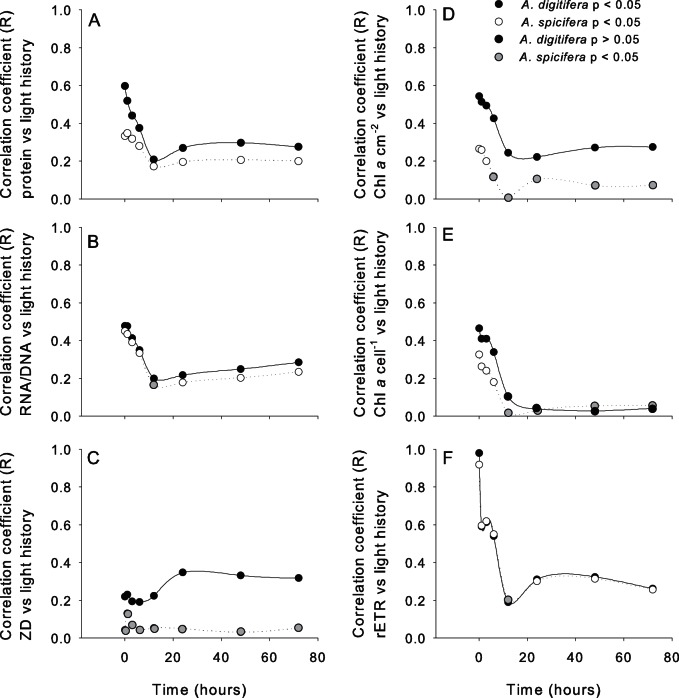
Correlation coefficient (R) between health indices and light history. Determined for A) protein concentration (mg mgDNA-1), B) RNA/DNA ratio, C) zooxanthellae density (cells cm^−2^), D) Chlorophyll *a* per surface area (mg cm^−2^), E) Chlorophyll *a* per cell (mg cell-1) and F) relative electron transport rate (rETR) (Based on DISTLm).

Correlations between light and health indices showed that light measurements taken at the time of sampling showed highest correlations for protein concentration, RNA/DNA ratios, rETR and Chl *a* concentration (Fig. 3). However, when light was integrated over the previous three days from the time of sampling, light was significantly positively correlated with protein concentration, RNA/DNA ratio and rETR for both species and with Chl *a* per surface area for *A. digitifera.* For *A. digitifera,* zoxanthellae density was correlated with light intensity one or more days prior to zooxanthellae sampling. However, for *A. spicifera* zooxanthellae density did not show any significant correlation with light. Overall, correlations coefficient for protein concentration, zooxanthellae density and Chl *a* concentration with light history were weaker for *A. spicifera* then *A. digitifera* (Fig. 3). Yield values only showed correlations with immediate light intensity (*A. digitifera*: r = 0.24, p<0.05; *A. spicifera*: r = 0.26, p<0.05).

### Diel Patterns of Physico-chemical Factors

**Figure 4:**
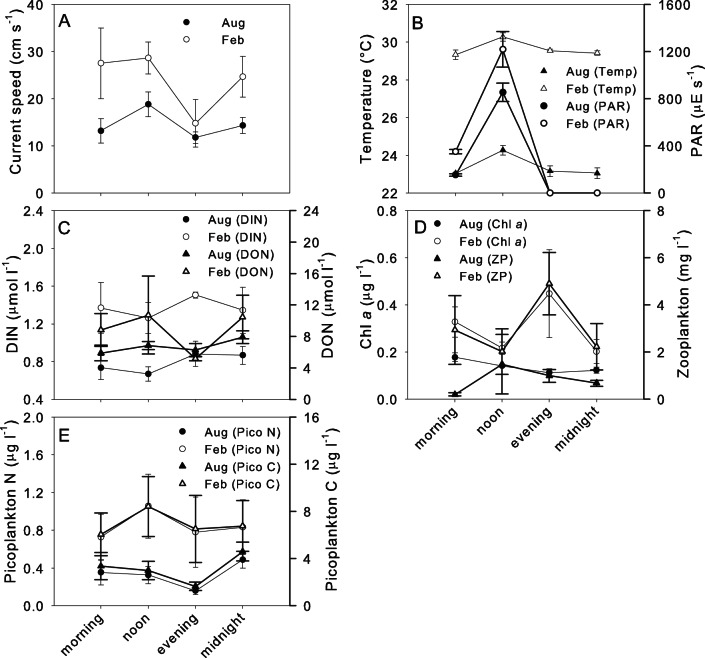
Diel patterns for physico-chemical parameters at Ningaloo Reef. Patterns in (A) current speed (B) temperature and light intensity, (C) dissolved inorganic nitrogen (DIN) and organic nitrogen (ON), (D) Chlorophyll *a* concentrations and zooplankton and (E) picoplankton carbon and nitrogen content at station 4 during August 2010 and February 2011. Data are means ± SE (n = 4).

In general, averaged values for all physico-chemical factors were lower in winter than in summer (Fig. 4). In winter 2010 and summer 2011, current speeds were highest at noon and lowest in the evening during. Similar patterns occurred for light and temperature during winter and summer, with highest values occurring at noon and lowest values occurring in the evening and at midnight. Picoplankton concentrations declined slightly in the evening in winter but showed comparable values throughout the day in summer. In summer, Chl *a* concentration and zooplankton showed similar patterns with lowest concentrations occurring at noon and highest concentrations in the evening while in winter Chl *a* concentration declined throughout the day and zooplankton peaked at noon. Highest DIN concentration was found in the evening for both seasons while ON showed lowest values in the evening for summer and no real change throughout the day in winter (Fig. 4).

## Discussion

### Temporal Changes in Health Indices and Suitability of Indicators for Monitoring

This is the first study to our knowledge, which determines how sensitive different metabolic and autotrophic indices are to the impact of long-term (seasonal) and short-term (diel and daily) changes in environmental factors. Previous work [30] reviewed the suitability of different health indices for projects monitoring coral health based on a variety of studies, but no study so far investigated the full range of metabolic indices (protein concentration, RNA/DNA ratio) and autotrophic indices (zooxanthellae density and pigment concentration, yield and relative electron transport rate) simultaneously. Here we determine the diel, daily and seasonal patterns in the fluctuation of multiple indices. Since light is important for all coral indices even though light may not be their proximal driver [38], we also determined how long it takes for health indices to react to light for a more sophisticated interpretation of diel, and daily, as well as seasonal, changes.

#### Protein concentration

Our results suggest that protein concentration is relatively stable on a diel basis, suggesting that this index could be used primarily to determine changes in seasonal and long-term coral health. This is in agreement with previous work which showed that protein concentration only shows detectable changes after weeks or months of perturbation, generally associated with changes in particulate food availability [16], [57], [58] or extreme temperatures [59], [60]. The precise time it takes to build-up and use protein reserves seems to be species-specific and depends on individual metabolic rates [59], [60]. While metabolic rates of *A. spicifera* were generally higher than of *A. digitifera* [32], no recurrent diel or daily patterns were observed for either species. Significant correlations between protein concentration and short-term light exposure (seconds – minutes), as well as (slightly weaker) correlations between integrated light exposure over the previous several days suggest that protein content does in fact respond to light, but that corals effectively integrate energy across diel fluctuations in light intensity, such that heterotrophic processes including protein synthesis, remain relatively stable. For example, coral’s protein synthesis is often dependent on DIN uptake, a light dependent process, which has been shown to express diel patterns [61]. In fact, overall in our study, nitrogen was a primary physico-chemical predictor for protein concentration in *A. digitifera* on a diel basis and for A. *spicifera* protein synthesis was correlated with light across seasons [38]. Positive correlations of protein concentration with light (seconds to days) might also be related to changes in the translocation rates of photosynthetic products from the zooxanthellae to the coral host which can be as rapid as 15 minutes, but might take 48 hours, at which time the products are finally integrated into coral tissue [31]. Overall, it is likely that a variety of physico-chemical factors varying on a diel, daily and seasonal basis light, nitrogen concentrations and zooplankton concentrations [38] will influence changes in protein concentrations in corals; however these changes are integrated via the longer metabolic time lines for protein synthesis. This index therefore emerges as a robust choice for the measurement of medium to long-term changes in coral health.

#### RNA/DNA ratio

RNA/DNA ratio showed significant differences between species, between seasons, between days and times of the day. This index is therefore an appropriate indicator for short as well as long-term changes in metabolic rates of corals. Because daily and diel variations are smaller than seasonal differences, the RNA/DNA ratio also provides an index of coral health that integrates appropriately across time scales. Seasonal changes in RNA/DNA ratio have been seen for corals [14] as well as changes within days when transplanted to different depths [42]. Diel patterns in our study were only recorded when data between species was pooled perhaps due a too small sample size given the intraspecific variation. In general, there is a clear response of RNA/DNA ratio to diel physiological fluctuations [30] as described for other species such as molluscs [23] and fish [22]. In our study, both species showed a winter pattern of lowest RNA/DNA ratio at noon and highest at midnight, while summer patterns were more variable, with highest RNA/DNA ratio in the day time, possibly since physico-chemical factors showed stronger diel fluctuations in summer then winter. Given the apparent importance of light, plankton concentrations and nitrogen concentrations in driving RNA/DNA ratios [14], [38], we suggest that this reflects corals’ reliance on photosynthates transferred from the zooxanthellae to the coral host, as well as on energy gained through heterotrophic feeding. Short-term (seconds to minutes) light variation showed the strongest correlation with RNA/DNA ratio for both *Acropora* species but also integrated light level from up to three previous days were correlated with the RNA/DNA ratio. However, the lack of correlation between RNA/DNA ratio and rETR, an indicator for photosynthetic activity [12], is contradictory to the hypothesis that diel variations in RNA/DNA ratios are driven by photosynthetically derived carbon. However, even though no clear correlations exist, diel variations in photosynthesis are likely to be at least partly responsible for diel changes in RNA/DNA ratio potentially due to a time lag effect of the integration of translocation products into the tissue [31] or an overestimation of rETR [12]. Plankton and nitrogen concentration can also trigger diel and daily variations in coral health, since diel cycles are often set by the availability of demersal plankton food [17], [18] and alternate sources of nitrogen [19] which can change on a daily as well as seasonal basis. This time-lag in feeding can be observed in the dynamics of coral food vacuoles, which appear in digestive cells within 2 hours of feeding and subsequently decrease in number per digestive cell and as the percentage of digestive cells with food vacuoles, only after 5–7 hours post-feeding [9]. Overall, RNA/DNA ratio can be used as a short-term as well as long-term indicator even though further studies are needed. When used in monitoring projects it has to be kept in mind though that RNA/DNA measurements should be taken at the same time each day to make data comparable.

#### Zooxanthellae densities and pigments

In general, zooxanthellae densities in our study fell within the range of previous studies, ranging between 0.5×10^6^ and 5×10^6^ cells per cm^2^ [29], [62], [63]. Zooxanthellae densities in corals in our study were surprisingly resistant to diel and daily changes in irradiance. Diel changes in zooxanthellae densities have been observed for some species due to synchronous division and degradation over a diel cycle [8], [9]. However, this diel cycle in corals seems highly species specific and not always present [40], [64], [65]. The results in our study suggest that if such cycles occur in zooxanthellae division, other processes regulating cell concentrations are likely to be operating at the same time, such as degradation [66], digestion and extrusion [65], resulting in no significant change in zooxanthellae density.

The strongest correlation of incident light with zooxanthellae density occurred more than 24 hours prior to sampling in *A. digitifera*. Previous studies have shown that it takes 24 hours for zooxanthellae densities to be modified [67], since algal division rate is set to more than 24 hours prior to actual cytokinesis [9]. Rhythmical changes in proliferation of zooxanthellae with periods of 3–6 days can certainly occur in addition to daily proliferation periodicity [68] due to changes in light [9], as well as local supply of zooplankton and nutrient concentrations [9], [69]. The fact that for *A. spicifera*, changes in zooxanthellae density do not seem to be directly driven by light history within either hours or days, suggests that zooxanthellae density is driven primarily by other factors, such as nitrogen uptake [38]. Our results suggest that for certain species such as *A. digitifera* zooxanthellae densities can be used to describe long-term (seasonal) changes in coral health. This is in agreement with other studies which also observed seasonal changes in zooxanthellae densities [28], [29].

Daily changes in physico-chemical factors such as temperature, light and nutrient concentrations might have been not large enough to result in measurable changes in zooxanthellae density. Seasonal environmental variation is much greater, resulting in larger variation in symbiont densities. Temperature, nutrients and light were all observed to be important in driving measurable variation in coral health indices [38].

We found no seasonal or diel difference for Chl *a* per unit coral surface area, or for Chl *a* per zooxanthellae. Our measured daily variations suggest Chl *a* can be a good indicator for short-term (days) changes in physico-chemical impacts on coral health. Daily changes in light intensity have also been known to impact coral Chl *a* concentrations [70], but no significant diel patterns were observed in our study or a previous study [5]. Our results do indicate that short term light levels (within hours) have the strongest impact on Chl *a* concentrations, suggesting there are no real time-lag effects. In regard to seasonal changes no significant variation was detected in this study in contrast with previous studies that have shown significant variations in Chl *a* concentration throughout the year in the studied species [28], [29], [71]. Thus Chl *a* might be suitable to describe seasonal changes in physico-chemical factors in case these changes are big enough, however it should be kept in mind that daily changes might also influence these patterns.

#### Effective quantum yield and relative ETR

Diel patterns in photosynthetic yield of *A. spicifera* are in accordance with previous studies showing highest values at night, a decrease in the morning, low values around noon and an increase during afternoon towards evening [10], [72], [73]. Down-regulation is attributed to dynamic photo-inhibition mediated by non-photochemical quenching in the reaction centre and antenna pigment bed [44], [73]. Yield depends on daily occurrence of peak solar radiation and is reversible and photo-protective [74]. The slightly different pattern for *A. digitifera*, with lowest values in the morning might be due to different genetic types of Symbiodinium [75], [76], photo-protection trait in the tissue [77] and tissue thickness [78]. In general yield values between 0.3 and 0.6 were in the range of previous studies [11], [79]. The fact that overall no species-specific differences occurred, suggest that both *Acropora* species’ photosystems react in similar ways to variations in light levels. However, yield values showed only a low correlation with short-term light history (R = 0.24 and 0.26) suggesting that other physico-chemical factors are more important for changes in yield. Despite previous studies which showed seasonal changes in yield values due to temperature [27], [80] our study did not observe seasonal variations even though temperature difference varied between 23 and 31**°**C between winter and summer. Overall for the studied *Acropora* species yield does not seem to be a good indicator for determining any seasonal differences, however yield appears more appropriate indicator for short-term changes since it reacts quickly to variations in light intensity. However since our study fell within a La Niña year and light values during summer were lower than normal [38] further studies are needed to determine seasonal changes. In addition when used for short-term monitoring projects light intensities are needed and samples should be taken at the same time to make data comparable.

Seasonal, daily and diel changes in rETR were clearly observed in our study, suggesting this measure was a good short-term and long-term indicator of coral health. Higher values at noon than in the morning are in the same range as those seen in previous studies [12], [72] for both species. Higher values during summer than in winter are also in accordance with previous work [27]. Short-term light history had an overwhelming correlation with changes in rETR while also light from previous day showed a smaller impact. Our values are in the range of other studies for morning (50–150) and midday (100–200) [12] as well as with seasonal studies [27]. Rates are influenced by phylotype and physiology (colour and tissue thickness of the coral species) of resident *Symbiodinium* sp., colony morphology, animal behaviour (polyp extension and contraction) as well as the light history of a particular location from which fluorescence measurements are taken [72], [81]. However, photosynthetic productivity can be overestimated when interpreted only through rETR [12] since rETR is non-linearly correlated with primary productivity and the relationship between biochemical and energetic assays of photosynthesis is influenced by photo-acclimatisation state of individual colonies [12]. Thus the photosynthetic rates we estimated at noon when PAR was highest might be an overestimation.

### Summary/Conclusion

Overall, we conclude that zooxanthellae density and protein concentrations are good long-term indicators of changes in coral health since they express seasonal but not diel or daily changes, however species-specific variations must be taken into account. RNA/DNA ratios and rETR can be used as long-term as well as short-term indices changes in coral health. However diel changes in RNA/DNA ratios remain unresolved. Chl *a* concentration in coral tissue is a better indicator for daily changes but should be used carefully to describe seasonal changes in coral health, while photosynthetic yield is good indicator to describe diel and daily variations for the two studied *Acropora* species at Ningaloo Reef. The fact that Chl *a* per cell is positively correlated with yield suggests that photo-adaptation processes in the antennae and PSII are related [82]. Species-specific differences in the dependence of light, in particular in regard to autotrophic indices, suggest that *A. digitifera* relies more strongly on autotrophy, which is in accordance with our earlier work [38] and most likely related to differences in zooxanthellae clades [83], differences in morphology [84], photo-protection trait in the tissue [77] and/or tissue thickness [78]. More studies are needed for a better interpretation of diel changes in RNA/DNA ratios as well as seasonal changes during seasons which do not fall in La Niña years. This is particularly important since our study reflects the normal variation during winter within the corals, however summer values were unexpected as shown previously [32].

## Supporting Information

Table S1 **Four-factorial PERMANOVA for health indices of two** ***Acropora*** **species.**
(DOCX)

Table S2 **Pair-wise correlations (p-values) for diel variations of A) effective quantum yield and B) relative electron transport rate.**
(DOCX)

Table S3 **Pair-wise correlations (p-values) for diel variations for RNA/DNA ratios.**
(DOCX)
