# Supplementary material for: Temporal Variations in Metabolic and Autotrophic Indices for Acropora digitifera and Acropora spicifera – Implications for Monitoring Projects
Source: PLoS One. 2013 May 16;8(5):e63693. doi: 10.1371/journal.pone.0063693 (PMC3655939; doi:10.1371/journal.pone.0063693)
Supplement: Table S1 — Four-factorial PERMANOVA for health indices of two Acropora species. (DOCX) [file pone.0063693.s001.docx]

Table S1 Four-factorial PERMANOVA for health indices of two *Acropora* species.

| **Variable** | **Effect** | **Df** | **SS** | **MS** | **Pseudo-F** | **p-value** |
| --- | --- | --- | --- | --- | --- | --- |
| Protein | sp | 1 | 0.1818 | 0.1818 | 58.27 | 0.000 |
| (mg µgDNA^-1^) | se | 1 | 0.1925 | 0.1925 | 27.44 | 0.010 |
|  | di | 3 | 0.0249 | 0.0083 | 2.41 | 0.089 |
|  | da(se) | 7 | 0.0454 | 0.0065 | 1.62 | 0.143 |
|  | spxse | 1 | 0.0499 | 0.0499 | 16.31 | 0.006 |
|  | spxdi | 3 | 0.0190 | 0.0063 | 2.12 | 0.123 |
|  | sexdi | 3 | 0.0155 | 0.0052 | 1.60 | 0.225 |
|  | spxda(se) | 7 | 0.0199 | 0.0028 | 0.71 | 0.658 |
|  | da(se)xdi | 16 | 0.0518 | 0.0032 | 0.81 | 0.671 |
|  | spxsexdi | 3 | 0.0187 | 0.0062 | 2.13 | 0.139 |
|  | spxda(se)xdi | 16 | 0.0468 | 0.0029 | 0.73 | 0.755 |
| RNA/DNA ratio | sp | 1 | 4.9404 | 4.9404 | 81.83 | 0.000 |
|  | se | 1 | 2.6178 | 2.6178 | 4.87 | 0.027 |
|  | di | 3 | 0.0559 | 0.0186 | 0.13 | 0.939 |
|  | da(se) | 7 | 3.4890 | 0.4984 | 10.53 | 0.000 |
|  | spxse | 1 | 0.3696 | 0.3696 | 6.34 | 0.041 |
|  | spxdi | 3 | 0.3221 | 0.1074 | 1.65 | 0.206 |
|  | sexdi | 3 | 0.6205 | 0.2068 | 1.43 | 0.270 |
|  | spxda(se) | 7 | 0.3815 | 0.0545 | 1.15 | 0.345 |
|  | da(se)xdi | 16 | 2.2962 | 0.1435 | 3.03 | 0.000 |
|  | spxsexdi | 3 | 0.4345 | 0.1448 | 2.29 | 0.122 |
|  | spxda(se)xdi | 16 | 1.0074 | 0.0630 | 1.33 | 0.186 |
| ZD (cells cm^-2^) | sp | 1 | 4.6373 | 4.6373 | 51.34 | 0.000 |
|  | se | 1 | 0.6731 | 0.6731 | 4.94 | 0.041 |
|  | di | 3 | 0.3619 | 0.1206 | 0.63 | 0.599 |
|  | da(se) | 7 | 0.8935 | 0.1277 | 0.91 | 0.504 |
|  | spxse | 1 | 0.4478 | 0.4478 | 5.12 | 0.058 |
|  | spxdi | 3 | 0.3857 | 0.1286 | 1.26 | 0.306 |
|  | sexdi | 3 | 0.2964 | 0.0988 | 0.51 | 0.675 |
|  | spxda(se) | 7 | 0.5791 | 0.0827 | 0.59 | 0.756 |
|  | da(se)xdi | 16 | 3.0960 | 0.1935 | 1.38 | 0.163 |
|  | spxsexdi | 3 | 0.0394 | 0.0131 | 0.13 | 0.939 |
|  | spxda(se)xdi | 16 | 1.6451 | 0.1028 | 0.73 | 0.753 |
| Chl *a* (µg cm^-2^) | sp | 1 | 1.5322 | 1.5322 | 91.98 | 0.000 |
|  | se | 1 | 0.0030 | 0.0030 | 0.02 | 0.921 |
|  | di | 3 | 0.1964 | 0.0655 | 0.84 | 0.473 |
|  | da(se) | 7 | 2.9806 | 0.4258 | 6.89 | 0.000 |
|  | spxse | 1 | 0.8372 | 0.8372 | 51.33 | 0.000 |
|  | spxdi | 3 | 0.2583 | 0.0861 | 1.28 | 0.296 |
|  | sexdi | 3 | 0.1697 | 0.0566 | 0.86 | 0.483 |
|  | spxda(se) | 7 | 0.1058 | 0.0151 | 0.24 | 0.974 |
|  | da(se)xdi | 16 | 1.0489 | 0.0656 | 1.06 | 0.405 |
|  | spxsexdi | 3 | 0.0657 | 0.0219 | 0.32 | 0.801 |
|  | spxda(se)xdi | 16 | 1.0935 | 0.0683 | 1.11 | 0.352 |
| Chl *a* (µg cell^-1^) | sp | 1 | 4.939 x 10^-12^ | 4.9398 x 10^-12^ | 1.69 | 0.238 |
|  | se | 1 | 18.915 x 10^-12^ | 18.915 x 10^-12^ | 1.48 | 0.269 |
|  | di | 3 | 25.822 x 10^-12^ | 8.607 x 10^-12^ | 1.88 | 0.167 |
|  | da(se) | 7 | 83.853 x 10^-12^ | 11.979 x 10^-12^ | 3.21 | 0.005 |
|  | spxse | 1 | 3.835 x 10^-12^ | 3.835 x 10^-12^ | 1.34 | 0.289 |
|  | spxdi | 3 | 0.813 x 10^-12^ | 0.271 x 10^-12^ | 0.13 | 0.936 |
|  | sexdi | 3 | 17.490 x 10^-12^ | 5.830 x 10^-12^ | 1.38 | 0.291 |
|  | spxda(se) | 7 | 20.010 x 10^-12^ | 2.858 x 10^-12^ | 0.77 | 0.617 |
|  | da(se)xdi | 16 | 67.451 x 10^-12^ | 4.216 x 10^-12^ | 1.13 | 0.337 |
|  | spxsexdi | 3 | 5.153 x 10^-12^ | 1.718 x 10^-12^ | 0.55 | 0.652 |
|  | spxda(se)xdi | 16 | 49.786 x 10^-12^ | 3.112 x 10^-12^ | 0.83 | 0.644 |
| Yield (ΔF/Fm’) | sp | 1 | 0.0168 | 0.0168 | 3.71 | 0.105 |
|  | se | 1 | 0.0639 | 0.0639 | 1.63 | 0.234 |
|  | di | 3 | 0.2183 | 0.0728 | 3.10 | 0.045 |
|  | da(se) | 7 | 0.2287 | 0.0327 | 11.40 | 0.000 |
|  | spxse | 1 | 0.0005 | 0.0005 | 0.23 | 0.651 |
|  | spxdi | 3 | 0.0217 | 0.0072 | 1.54 | 0.226 |
|  | sexdi | 3 | 0.0676 | 0.0225 | 1.04 | 0.410 |
|  | spxda(se) | 7 | 0.0273 | 0.0039 | 1.36 | 0.233 |
|  | da(se)xdi | 13 | 0.2812 | 0.0216 | 7.55 | 0.000 |
|  | spxsexdi | 3 | 0.0051 | 0.0017 | 0.37 | 0.767 |
|  | spxda(se)xdi | 13 | 0.0594 | 0.0046 | 1.59 | 0.095 |
| rETR | sp | 1 | 0.0515 | 0.0515 | 1.20 | 0.311 |
|  | se | 1 | 15.5840 | 15.5840 | 37.14 | 0.000 |
|  | di | 3 | 826.5400 | 275.5100 | 388.87 | 0.000 |
|  | da(se) | 7 | 2.4288 | 0.3470 | 17.74 | 0.000 |
|  | spxse | 1 | 0.0029 | 0.0029 | 0.15 | 0.718 |
|  | spxdi | 3 | 0.2913 | 0.0971 | 2.77 | 0.068 |
|  | sexdi | 3 | 3.3550 | 1.1183 | 1.62 | 0.236 |
|  | spxda(se) | 7 | 0.2695 | 0.0385 | 1.97 | 0.064 |
|  | da(se)xdi | 13 | 8.9533 | 0.6887 | 35.22 | 0.000 |
|  | spxsexdi | 3 | 0.0388 | 0.0129 | 0.39 | 0.771 |
|  | spxda(se)xdi | 13 | 0.4322 | 0.0332 | 1.70 | 0.068 |

Protein concentration, RNA/DNA ratio, zooxanthellae density (ZD), Chl a concentration per surface area and per cell, yield and relative electron transport rate (rETR) were compared for 2 species (sp) (A. digitifera and A. spicifera), 2 seasons (se) (August 2010 (winter) and February 2011(summer)), over 4 days each season (da) and 4 different times a day (di) (morning, noon, evening, midnight) at Sandy Bay, Ningaloo Reef. Species, season and time of the day were fixed effects, while days are nested within seasons. PERMANOVA was based on euclidean distance after log-transformation. Type I (sequential) sum of squares was used with permutation of residuals under a reduced model (9999 permutations).
