# Supplementary material for: Temporal Variations in Metabolic and Autotrophic Indices for Acropora digitifera and Acropora spicifera – Implications for Monitoring Projects
Source: PLoS One. 2013 May 16;8(5):e63693. doi: 10.1371/journal.pone.0063693 (PMC3655939; doi:10.1371/journal.pone.0063693)
Supplement: Table S2 — Pair-wise correlations (p-values) for diel variations of A) effective quantum yield and B) relative electron transport rate. (DOCX) [file pone.0063693.s002.docx]

Table S2. Pair-wise correlations (p-values) for diel variations of A) effective quantum yield and B) relative electron transport rate.

| Season | Aug-10 | | | Feb-11 | | |
| --- | --- | --- | --- | --- | --- | --- |
| Days | 1 | 2 | 3 | 1 | 2 | 3 |
| **A Effective quantum yield** | | | | |  |  |
| midnight, noon | 0.69 | **0.003** | 0.643 | 0.699 | **0.012** | **0.021** |
| midnight, morning | **0.002** | **0.016** | 0.52 | 0.471 | **0.01** | 0.07 |
| midnight, evening | **0.003** | **0.012** | 0.288 | 0.058 | 0.67 | e.n.m |
| noon, morning | **0.003** | **0.006** | 0.829 | 0.325 | 0.77 | 0.626 |
| noon, evening | **0.009** | **0.003** | 0.769 | 0.109 | **0.006** | e.n.m |
| morning, evening | **0.029** | 0.858 | 0.952 | 0.058 | **0.003** | e.n.m |
| **B relative electron transport rate** | | | | | |  |
| midnight, noon | **0.003** | **0.002** | **0.001** | **0.003** | **0.002** | **0.003** |
| midnight, morning | **0.003** | **0.003** | **0.002** | **0.001** | **0.001** | **0.002** |
| midnight, evening | determinator both 0 | | | | | |
| noon, morning | **0.003** | 0.072 | **0.002** | **0.003** | **0.002** | **0.002** |
| noon, evening | **0.003** | **0.002** | **0.002** | **0.002** | **0.002** | e.n.m |
| morning, evening | **0.002** | **0.002** | **0.002** | **0.001** | **0.003** | e.n.m |

Values were pooled together for both *Acropora spicifera* and *Acropora digitifera*. Analysis was done with PERMANOVA (Primer). E.n.m = evening not measured.
