# Supplementary material for: Temporal Variations in Metabolic and Autotrophic Indices for Acropora digitifera and Acropora spicifera – Implications for Monitoring Projects
Source: PLoS One. 2013 May 16;8(5):e63693. doi: 10.1371/journal.pone.0063693 (PMC3655939; doi:10.1371/journal.pone.0063693)
Supplement: Table S3 — Pair-wise correlations (p-values) for diel variations for RNA/DNA ratios. (DOCX) [file pone.0063693.s003.docx]

Table S3. Pair-wise correlations (p-values) for diel variations for RNA/DNA ratios.

| **RNA/DNA ratio** | | | | | | | |
| --- | --- | --- | --- | --- | --- | --- | --- |
| Season | Aug-10 | | | | Feb-11 | | |
| Days | 1 | 2 | 3 | 4 | 1 | 2 | 3 |
| midnight, noon | 0.157 | 0.962 | **0.042** | **0.013** | **0.012** | 0.06 | **0.049** |
| midnight, morning | 0.31 | 0.996 | **0.036** | 0.27 | 0.479 | 0.027 | 0.391 |
| midnight, evening | 0.361 | 0.084 | 0.07 | 0.948 | 0.23 | 0.053 | e.n.m |
| noon, morning | 0.785 | 0.929 | 0.694 | **0.011** | 0.055 | 0.837 | **0.047** |
| noon, evening | 0.621 | 0.192 | 0.777 | **0.044** | 0.523 | 0.511 | e.n.m |
| morning, evening | 0.62 | 0.126 | 0.959 | 0.297 | 0.461 | 0.352 | e.n.m |

Values were pooled together for both *Acropora spicifera* and *Acropora digitifera*. Analysis was done with PERMANOVA (Primer). E.n.m = evening not measured.
